# Impact of long-term exposure to PM_2.5_ and temperature on coronavirus disease mortality: observed trends in France

**DOI:** 10.1186/s12940-021-00784-1

**Published:** 2021-09-06

**Authors:** Anastase Tchicaya, Nathalie Lorentz, Hichem Omrani, Gaetan de Lanchy, Kristell Leduc

**Affiliations:** Living Conditions Department, Luxembourg Institute of Socio-Economic Research, 11 Porte des Sciences, L-4366 Esch-sur-Alzette, Luxembourg

**Keywords:** COVID-19 mortality rate, Long-term exposure to PM_2.5_, Air pollution, Temperature, Spatial disparity, France

## Abstract

**Background:**

The outbreak of coronavirus disease (COVID-19) began in Wuhan, China in December 2019 and was declared a global pandemic on 11 March 2020. This study aimed to assess the effects of temperature and long-term exposure to air pollution on the COVID-19 mortality rate at the sub-national level in France.

**Methods:**

This cross-sectional study considered different periods of the COVID-19 pandemic from May to December 2020. It included 96 departments (or NUTS 3) in mainland France. Data on long-term exposure to particulate matter (PM_2.5_), annual mean temperature, health services, health risk, and socio-spatial factors were used as covariates in negative binomial regression analysis to assess their influence on the COVID-19 mortality rate. All data were obtained from open-access sources.

**Results:**

The cumulative COVID-19 mortality rate by department increased during the study period in metropolitan France—from 19.8/100,000 inhabitants (standard deviation (SD): 20.1) on 1 May 2020, to 65.4/100,000 inhabitants (SD: 39.4) on 31 December 2020. The rate was the highest in the departments where the annual average of long-term exposure to PM_2.5_ was high. The negative binomial regression models showed that a 1 μg/m^3^ increase in the annual average PM_2.5_ concentration was associated with a statistically significant increase in the COVID-19 mortality rate, corresponding to 24.4%, 25.8%, 26.4%, 26.7%, 27.1%, 25.8%, and 15.1% in May, June, July, August, September, October, and November, respectively. This association was no longer significant on 1 and 31 December 2020. The association between temperature and the COVID-19 mortality rate was only significant on 1 November, 1 December, and 31 December 2020. An increase of 1 °C in the average temperature was associated with a decrease in the COVID-19-mortality rate, corresponding to 9.7%, 13.3%, and 14.5% on 1 November, 1 December, and 31 December 2020, respectively.

**Conclusion:**

This study found significant associations between the COVID-19 mortality rate and long-term exposure to air pollution and temperature. However, these associations tended to decrease with the persistence of the pandemic and massive spread of the disease across the entire country.

**Supplementary Information:**

The online version contains supplementary material available at 10.1186/s12940-021-00784-1.

## Background

The outbreak of coronavirus disease (COVID-19) began in Wuhan, China in December 2019, and quickly spread worldwide. COVID-19 is a respiratory infection caused by severe acute respiratory syndrome coronavirus 2 (SARS-CoV-2). The World Health Organization (WHO) declared a state of global health emergency on 31 January 2020 [1] and the state of a pandemic on 11 March 2020. Several epidemiological studies have shown that air pollution increases the incidence of a wide range of diseases, mainly respiratory and cardiovascular diseases [2–6]. Exposure to air pollution is associated with vulnerability, including low socioeconomic status (SES), high-risk occupation, and pre-existing health problems [3, 6, 7]. People with low SES are more likely to work outdoors or in places with air pollution or extreme temperatures [5]. In contrast, very high-income groups tend to work indoors, which reduces their exposure [3, 5].

Furthermore, people with pre-existing health problems tend to be significantly affected by air pollution. For example, heart attack survivors are more likely to be readmitted to hospitals and have a very high mortality rate if they are exposed to long-term air pollution [6].

The association between long-term exposure to PM_2.5_ and mortality is well documented [2, 4, 8–12], with chronic exposure to PM_2.5_ most strongly associated with mortality attributable to ischemic heart disease, arrhythmia, heart failure, and cardiac arrest [2].

Most studies conducted since the beginning of the COVID-19 pandemic have shown that environmental factors such as air pollution and ambient temperature can be considered crucial mediators in COVID-19 spread and mortality [13–20]. An analysis of the spatial or geographic distribution of severe infections and deaths from COVID-19 highlighted that populations living in areas with high levels of air pollution were more likely to develop severe COVID-19 or die than other populations. The mechanisms by which these two environmental factors interact with COVID-19 are not well established. For example, Wang et al. [14] considered that PM_2.5_ could facilitate SARS-CoV-2 infection through overregulation of angiotensin-converting enzyme 2 (ACE2).

In France, in 2015, the annual mean PM_2.5_ concentration level was 11.9 μg/m^3^ and was responsible for 35,800 premature deaths and 624 years of life lost (7). The average concentration level of PM_2.5_ slightly above the 10 μg/m^3^ limit set by the WHO masks the spatial disparities in PM_2.5_ exposure and its impact on the COVID -19 spread and mortality. However, further investigations are needed.

As of 31 December 2020, France declared 44,456 cumulative deaths in hospitals due to COVID-19 (Ministry of Health/Directorate General of Health 2020). The spatial distribution of COVID-19 mortality was unequal across departments, but the determinants of unevenness were unknown.

Therefore, the present study aimed to assess the effects of long-term exposure to both air pollution and temperature on the COVID-19 mortality rate in mainland France, at different times of the evolution of COVID-19 pandemic. To the best of our knowledge, this study is the first to assess the combined associations of two environmental factors with COVID-19 mortality rate measured over a relatively large period at the sub-national level in France or in Europe. Further, this study measured the risk of COVID-19 mortality with regard to the PM_2.5_ concentration level by quartile to highlight the impact of inequalities in the long-term exposure to PM_2.5_ and its disparities in COVID-19 mortality rates observable at the sub-national level.

## Materials and Methods

### Design

This study cross-sectional analysis used data on COVID-19 mortality, environmental factors (PM2.5 and temperature), and socio-spatial factors measured at the level of the departments (NUTS 3). The cross-sectional analysis was performed on different dates during the pandemic (from 1 May to 31 December 2020) across 96 departments of mainland France. Two epidemic waves were observed during the study period, with the second wave emerging from September (Supplementary Material Fig. 1).

From this perspective, only the cumulative number of deaths changed, and all other independent variables characterising the different departments remained unchanged. One of the objectives was to understand the extent to which pre-existing conditions before the COVID-19 outbreak could play a significant role in the COVID-19 spread and severity observed in the country despite implementation of various control measures at the national level (such as lockdown, physical and social distancing, curfews, teleworking, travel restrictions, and wearing masks).

### Data sources and data

Several data sources were used in this study, and all the variables used are shown in Table 1 (the full dataset is publicly accessible) [21]. Cumulative data on COVID-19 deaths in the hospitals were obtained from the French Ministry of Health statistics.

**Table 1:** Variables, definitions and data sources

Variables	Definition	Data sources	Data year	Level considered
COVID-19 death rate in hospitals	Number of cumulated COVID-19 deaths in hospitals expressed per 100 000 people	Ministry of Health https://www.data.gouv.fr/fr/datasets/donnees-hospitalieres-relatives-a-lepidemie-de-covid-19/	2020	Department
*Availability of healthcare services*				
Number of resuscitation beds	Number of resuscitation beds expressed per 100 000 people	Ministry of Health https://drees.solidarites-sante.gouv.fr	2018	Department
Number of intensive care beds	Number of intensive care beds expressed per 100 000 people	Ministry of Health https://drees.solidarites-sante.gouv.fr	2018	Department
Physicians density	Number of physicians divided by the department area	Ministry of Health https://drees.solidarites-sante.gouv.fr	2018	Department
*Socio-spatial factors*				
%People aged 60 +	Proportion of the population 60 years of age or older	Eurostat	2019	Department
%Males	Proportion of male sex in population	Eurostat	2019	Department
%Unemployment	Proportion of adults unemployed adults	Eurostat	2019	Department
%Urban population	Proportion of people living in the greater urban areas (per cent)	FNORS/STATISS 2019, version of May 18, 2020	2016	Department
Rate of poverty	Rate of monetary poverty (per cent)	FNORS	2016	Department
Population density	Number of people by square kilometer	Eurostat	2019	Department
Population size	Size of population for each department (by thousand)	Eurostat	2019	Department
*Health risk factors*				
Standardized Prevalence rate of Diabetes	Standardized prevalence rate of pharmacologically treated diabetes (all types) (%)	https://www.santepubliquefrance.fr/maladies-et-traumatismes/diabete	2018	Department
*Environmental factors*				
PM_2.5_	Particle matter 2.5 (micro g / m3)	Global Ozone Monitoring Experiment (GOME)	1999–2016	Department
Temperature	Annual average of temperature (°C)	World climate https://www.worldclim.org	2001–2012	Department

NB: The rate is standardized according to age, the reference population being the whole of France in the 2006 population census

Data on environmental factors, including exposure to PM_2.5_ and air temperature, were obtained from the Global Ozone Monitoring Experiment and the World Climate, respectively (Table 1). Air pollution (i.e. PM_2.5_) data were downloaded from the NASA-Socioeconomic Data and Applications Centre. Temperature data were obtained from WorldClim-Version2. Initially, both variables had a resolution of 1 km × 1 km (or 1 km^2^), but later they were averaged and aggregated (using a mean operator) to obtain long-term exposure to air pollution (1999–2016) and temperature (2001–2012) at the departmental level (NUTS 3) in mainland France.

Data on the availability of health services (number of resuscitation beds, number of intensive care beds, and medical density) at the departmental level were mainly extracted from the Direction of Research, Studies, Evaluation and Studies.

Data on socio-spatial factors, including demographic, socio-economic, and spatial characteristics, were obtained from the European Statistical Office and the National Federation of Regional Health Observatories (FNORS) (Supplementary Material Table 1). Data on the prevalence of diabetes were obtained from the FNORS.

### Measures

#### Outcome

The COVID-19 mortality rate in hospitals at the departmental level was the primary health outcome of this study. It is defined as the number of COVID-19 deaths per 100,000 people.

#### Covariates

##### Air pollution exposure (PM_2.5_)

The WHO limit is 10 μg/m^3^, but the EU guidelines indicate an annual mean air quality limit of 25 μg/m ^3^ for PM_2.5_ since 2015, which is 2.5 times higher than the WHO limit [22].

The annual average PM_2.5_ concentration levels from 1999 to 2016 were computed to assess the potential association between long-term exposure to air pollution and the COVID-19 mortality rate. This period of measurement (i.e. annual average of exposure to PM_2.5_) considered in this study (> 18 years) is longer than studies from Europe and USA [15–17, 23–25]. The annual average PM_2.5_ concentration level was then defined in quartiles, thus making it possible to classify the departments according to the degree of their long-term exposure to air pollution (Supplementary Material Table 2).

##### Temperature

The annual average temperature (expressed in °C) was obtained from the World Climate website, covering the 2001–2012 period. Temperature is considered an environmental driver of several outbreaks, such as influenza. Shi et al. [18] found that temperature had a significant effect on the incidence of COVID-19. The authors showed that the transmission rate, which was correlated with the real-time temperature data, decreased as the temperature increased, leading to a reduction in the size and infection rate of the outbreak.

In this study, the PM_2.5_ and temperature are the primary factors of interest as exposures, and the other variables were included in the design as potential confounders of the association COVID-19 mortality and PM_2.5_ and temperature.

##### Availability of health services

The availability of health services such as resuscitation and intensive care units and physicians represents the health system’s ability to manage patients with severe COVID-19. Here, it was considered in terms of the number of resuscitation and intensive care beds and medical density at the department level. These data were defined per 100,000 people.

##### Socio-demographic factors

In this study, the socio-demographic factors included demographic (proportion of people aged ≥ 60 and the proportion of males), socioeconomic (proportion of adult people unemployed and poverty rate), and spatial or environmental characteristics (population density and the proportion of people living in large urban areas). However, this choice is constrained by the availability of information. These factors need to be carefully considered when estimating the association between air pollution and the incidence and mortality of COVID-19 [26].

##### Prevalence of diabetes

Diabetes was common among individuals who developed severe COVID-19 or died from it [27, 28]. It could represent a serious risk factor for COVID-19 in areas with a high prevalence rate. This health risk factor was calculated as the age-standardized prevalence rate per 100,000 individuals.

### Statistical analysis

The characteristics of the dataset regarding the number of COVID-19 deaths, air pollution, health service availability, socio-spatial factors, and health status are described as mean, standard deviation, minimum, and maximum values. We explored the relationship between COVID-19 mortality with long-term PM_2.5_ exposure and temperature using correlation analysis. We analysed the geographic distribution of COVID-19 mortality rate at the department level depending on long-term exposure to PM_2.5_ grouped into quartiles. Next, we performed negative binomial regression analysis to examine whether long-term PM_2.5_ exposure and temperature were associated with the COVID-19 mortality rate, adjusted for several confounders (e.g. health service availability, health risk factors, and socio-demographic factors). We implemented nine separate models to estimate the effect of PM_2.5_ exposure and temperature on the COVID-19 mortality rate, adjusted for potential confounders mentioned above (number of intensive care and resuscitation beds per 100,000 people, medical density per 100,000 people, share of people aged 60 or more, share of males in population, unemployment, rate of poverty, proportion of people living in the great urban areas, population density, and standardized prevalence of diabetes). These nine models corresponded to nine monthly dates considered during the period covered in this study. The same long term PM_2.5_ average and temperature were used to estimate association with COVID-19 mortality in different months. Furthermore, we performed negative binomial regression analysis to investigate the magnitude of disparities in COVID-19 mortality rate between departments with higher PM_2.5_ exposure levels and departments with lower PM_2.5_ exposure levels based on quartiles, adjusted for all potential confounders. This alternative modelling allowed us to show the robustness of the relationships between long-term PM_2.5_ exposure and the associated risk of COVID-19 mortality. Note that long-term exposure to PM_2.5_ and temperature were used together in all regressions performed monthly. The other analyses were performed as a robustness test, such as unadjusted regression analyses, to evaluate these relationships [29]. Negative binomial regression models allowed us to consider over-dispersion, which is often present in count data [30]. Indeed, the negative binomial regression model makes it possible to relax the hypothesis of equality between the mean and the variance of the Poisson regression by introducing an additional parameter, which considers the dispersion of the count data. Besides, when the dispersion parameter is set to 1, the result is called the geometric distribution. Thus, geometric regression is a special case of negative binomial regression. Finally, the negative binomial regression was a suitable approach for our study because it is more flexible regarding the value of the dispersion parameter, and it often presents better performance criteria. The multiplication coefficients of the negative binomial regression models corresponded to mortality rate ratios, which were interpreted as the percentage increase (or decrease) in the COVID-19 mortality rate associated with each quartile of long-term average PM_2.5_ exposure compared to the first quartile [31].

The results were assessed at a significant threshold of error of 5% or 95% confidence interval (95% CI). The parameter estimates from the negative binomial models were exponentiated to define the COVID-19 mortality rate ratio.

In addition, COVID-19 mortality maps were created to show the spatial disparities of the COVID-19 pandemic across metropolitan France using a free and open source as *PhilCarto software (*http://philcarto.free.fr*)* and *Inkspace* software (https://inkspace.org).

All analyses were performed using SAS software (release 9.4; SAS Institute Inc., Cary, NC, USA).

## Results

The epidemiological situation observed in France until 31 December 2020 was worrying, and all the departments were not affected in the same way.

Table 2 presents the distribution characteristics of the parameters used in this study for each department in France.

**Table 2:** Description of the study’s characteristics, including Covid-19 deaths on 1 May and 31 December, 2020, by mean (standard deviation), minimum and maximum

	Mean (standard deviation)	Min	Max
COVID-19 deaths rate in hospitals (per 100,000):			
● 1 May 2020	19.8 (20.1)	1.3	106.3
● 31 December 2020	65.4 (39.4)	12.5	281.1
Annual Average long-term PM_2.5_ exposure from 1999 to 2016 (μg/m^3^)	10.1 (2.4)	6.0	16.3
Annual average temperature over 12 years from 2001 to 2012 (in °C)	10.7 (1.6)	4.7	14.4
Number of intensive care and resuscitation beds (per 100,000)	14.1 (7.7)	2.4	47.1
Medical density (per 100,000)	305.0 (89.3)	167.0	858.0
% People aged 60 or more	29.6 (4.8)	16.7	39.3
% Males	48.5 (0.5)	46.9	49.6
% Unemployment	7.9 (1.6)	4.8	13.3
Rate of poverty (per cent)	14.6 (3.1)	9.2	28.6
% Urban population (proportion of people living in the great urban areas)	71.2 (20.8)	0.1	100.0
Population density (inhab/square) (by thousand)	565.8 (2425.1)	14.8	20,459.7
Population size (by thousand)	676.0 (520.1)	76.3	2589.0
Standardized Prevalence of Diabetes at the department-level	4.8 (0.8)	3.2	7.9

As of 31 December 2020, the COVID-19 mortality rate in the department of metropolitan France was 65.4 per 100,000 inhabitants (standard deviation [SD] = 39.4) in hospitals. The annual average PM_2.5_ concentration level from 1999 to 2016 was 10.1% (SD = 2.4), and the annual average temperature from 2001 to 2012 was 10.7% (SD = 1.6). The average number of intensive care and resuscitation beds per 100,000 inhabitants was 14.1 (SD = 7.7), and the average medical density was 305.0 (SD = 89.3) per department.

The departments’ socio-spatial characteristics showed that 29.6% of the population were aged ≥ 60 years, 51.5% were women, 7.9% were unemployed, and 71.2% were living in large urban areas.

**Fig. 1:**
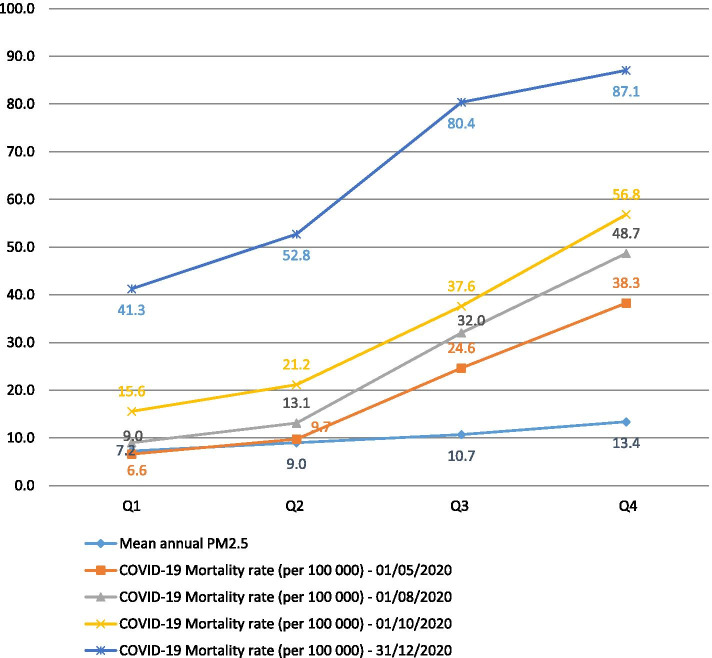
Distribution of the COVID-19 mortality rate and long-term PM_2.5_ exposure per quartile

The geographic distribution of COVID-19 mortality rates as a function of the annual average level of PM_2.5_, defined by quartiles, showed the existence of a gradient (see Fig. 1). At the start of the period, the mortality rate due to COVID-19 in the departments classified in quartiles 1, 2, 3, and 4 were 6.6, 9.7, 24.6 and 38.3 deaths per 100,000 inhabitants, respectively. At the end of the period, during the second wave of the pandemic, the mortality rates were 41.3, 52.8, 80.4, and 87.1 per 100,000 inhabitants, respectively.

**Fig. 2:**
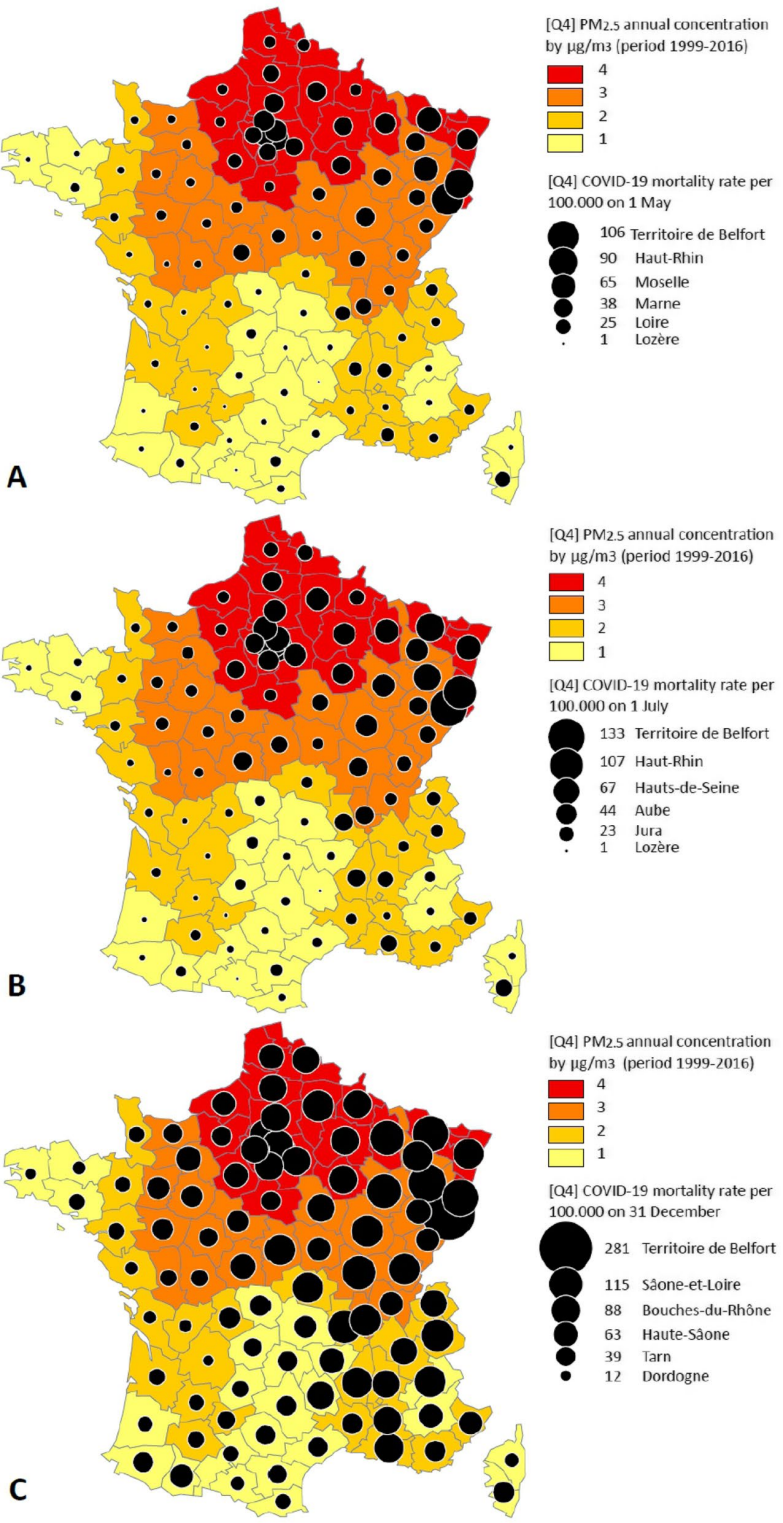
Geographical distribution of the COVID-19 mortality rate depending to quartile of PM_2.5_ concentration level by department on 1 May 2020 (**a**), 1 July (**b**), and 31 December 2020 (**c**)

The maps illustrate the dynamics of the spread of the COVID-19 pandemic across all departments compared to the annual average PM_2.5_ concentration over the long term (Fig. 2).

Figures 1 and 2 show that the COVID-19 mortality rate increased with the level of particle matters concentration.

Table 3 shows the estimates of the effects of long-term exposure to PM_2.5_, and temperature on the COVID-19 mortality rates on different dates. The COVID-19 mortality rate was significantly associated with long-term exposure to air pollution until 1 November, and was no longer so in December 2020. Thus, a concentration of 1 μg/m ^3^ of PM_2.5_, was associated with an increase in the COVID-19 mortality rate multiplied by a coefficient of 1.244, 1.264, 1.271, and 1.151, respectively, in May, July, September, and November 2020. The association between the COVID-19 mortality rate and temperature was not statistically significant until November 2020. The temperature was associated with a mortality rate multiplied by 0.905, 0.870, and 0.859, respectively, on 1 November, 1 December, and 31 December 2020. Multiplier coefficients were less than 1, which corresponded to a respective decrease in the mortality rate of 9.5%, 13.0%, and 14.1% for a 1 °C increase in the annual average temperature between the departments.

**Table 3:** Estimates effects of long-term PM_2.5_ exposure and Temperature on COVID-19 mortality rate using the negative binomial regression model, adjusted for all potential confounders (From 01 May to 31 December, 2020)

Independent variables	01 May	01 June	01 July	01 August	01 September	01 October	01 November	01 December	31 December
Annual average Long-term PM_2.5_ exposure (μg/m^3^)	1.244^a^ (1.083–1.428)	1.258^b^ (1.104–1.434)	1.264^b^ (1.111–1.439)	1.267^b^ (1.114–1.442)	1.271^b^ (1.118–1.444)	1.258^b^ (1.111–1.423)	1.151^b^ (1.036–1.279)	1.078 (0.985–1.180)	1.090 (0.999–1.190)
Annual average Temperature over 12 years	0.916 (0.829–1.012)	0.913 (0.832–1.002)	0.913 (0.832–1.001)	0.914 (0.834–1.002)	0.917 (0.837–1.004)	0.921 (0.844–1.005)	0.905^b^ (0.840–0.974)	0.870^b^ (0.816–0.927)	0.859^b^ (0.807–0.914)
Number of intensive care and resuscitation beds (per 100,000)	1.025^a^ (1.002–1.049)	1.020 (0.999–1.042)	1.019 (0.998–1.041)	1.018 (0.997–1.040)	1.018 (0.997–1.039)	1.015 (0.995–1.036)	1.011 (0.994–1.029)	1.003 (0.988–1.018)	1.006 (0.991–1.020)
Medical density (per 100,000)	0.998 (0.995–1.000)	0.999 (0.996–1.001)	0.999 (0.997–1.001)	0.999 (0.997–1.001)	0.999 (0.997–1.001)	1.000 (0.997–1.002)	1.000 (0.998–1.002)	1.001 (0.999–1.002)	1.001 (0.999–1.002)
% People aged 60 or more	1.088^b^ (1.021–1.158)	1.096^b^ (1.032–1.163)	1.097^b^ (1.034–1.164)	1.100^b^ (1.037–1.167)	1.097^b^ (1.035–1.163)	1.096^b^ (1.036–1.159)	1.046 (0.999–1.095)	1.037 (0.997–1.080)	1.039 (0.999–1.080)
% Males	1.467 (0.995–2.164)	1.521^a^ (1.050–2.203)	1.557^a^ (1.079–2.248)	1.589^a^ (1.102–2.291)	1.585^a^ (1.103–2.277)	1.571^a^ (1.106–2.233)	1.482^a^ (1.098–1.999)	1.331^a^ (1.025–1.730)	1.310^a^ (1.015–1.690)
% Unemployment	1.011 (0.872–1.173)	0.996 (0.866–1.145)	0.997 (0.868–1.145)	0.997 (0.868–1.145)	1.001 (0.873–1.148)	0.974 (0.854–1.110)	0.987 (0.883–1.105)	0.989 (0.897–1.091)	0.990 (0.899–1.090)
Rate of Poverty (per cent)	0.999 (0.904–1.104)	1.006 (0.916–1.105)	1.009 (0.920–1.107)	1.010 (0.921–1.108)	1.009 (0.921–1.106)	1.026 (0.940–1.120)	1.023 (0.948–1.103)	1.018 (0.953–1.087)	1.017 (0.954–1.084)
% Urban population (proportion of people living in the great urban areas)	1.021^b^ (1.006–1.037)	1.021^b^ (1.007–1.036)	1.021^b^ (1.007–1.036)	1.022^b^ (1.008–1.037)	1.021^b^ (1.007–1.036)	1.023^b^ (1.009–1.036)	1.013^a^ (1.002–1.024)	1.011^a^ (1.002–1.020)	1.008 (0.999–1.017)
Population density (inhab/square)	1.000 (1.000–1.000)	1.000 (1.000–1.000)	1.000 (1.000–1.000)	1.000 (1.000–1.000)	1.000 (1.000–1.000)	1.000 (1.000–1.000)	1.000 (1.000–1.000)	1.000 (1.000–1.000)	1.000 (1.000–1.000)
Standardized Prevalence of Diabetes (%)	1.160 (0.772–1.742)	1.136 (0.775–1.666)	1.138 (0.779–1.661)	1.134 (0.777–1.653)	1.125 (0.775–1.633)	1.126 (0.785–1.614)	1.187 (0.871–1.619)	1.216 (0.930–1.589)	1.188 (0.916–1.542)
*Criteria for assessing goodness of fit:*									
*Deviance*	*1.1594*	*1.1835*	*1.1859*	*1.1882*	*1.1841*	*1.1897*	*1.1574*	*1.171*	*1.1661*
*Scaled Deviance*	*1.1594*	*1.1835*	*1.1859*	*1.1882*	*1.1841*	*1.1897*	*1.1574*	*1.171*	*1.1661*
*Pearson Chi-Square*	*1.3409*	*1.2943*	*1.2899*	*1.2881*	*1.2894*	*1.2474*	*1.2029*	*1.1794*	*1.2021*
*Scaled Pearson X2*	*1.3409*	*1.2943*	*1.2899*	*1.2881*	*1.2894*	*1.2474*	*1.2029*	*1.1794*	*1.2021*
*BIC (smaller is better)*	*731.9157*	*765.3375*	*771.5867*	*774.6766*	*776.8832*	*784.2321*	*805.7196*	*879.2935*	*924.3351*

(^a^) Significance at 5%, (^b^) Significance at 1%

Furthermore, the unadjusted regression analyses assessing the relationships between exposure to PM_2.5_ and COVID-19 mortality rate or temperature and COVID-19 mortality rate showed significant associations on all the considered periods in this study (supplementary material Table 3).

Table 4 shows results of the PM_2.5_ concentration level defined in the quartile. The risk of death from COVID-19 was higher in departments with a PM_2.5_ concentration level equal to or greater than the third quartile, compared to those located in the first quartile. For example, departments with levels of PM_2.5_ exposure in the third quartile had 2.408, 2.295, 1.624, and 1.469 times greater risk of death from COVID-19 than departments placed in the first quartile, respectively on 1 May, 1 August, 1 November, and 31 December 2020. Note the absence of a statistically significant difference in the mortality rate between the departments with PM_2.5_ concentration level belonging to the first and the second quartile.

**Table 4:** Estimates effects of long-term PM_2.5_ exposure in quartile and Temperature on COVID-19 mortality rate using the negative binomial regression model, adjusted for all potential confounders (From 01May to 31 December, 2020)

Independent variables	01 May	01 June	01 July	01 August	01 September	01 October	01 November	01 December	31 December
Annual average Long-term PM_2.5_ exposure (μg/m^3^) in quartile (ref. = Q1 (7.2 μg/m^3^))									
Q2 (9.0 μg/m^3^)	1.134 (0.758–1.699)	1.144 (0.779–1.680)	1.134 (0.775–1.660)	1.127 (0.771–1.649)	1.126 (0.773–1.640)	1.144 (0.796–1.643)	1.105 (0.807–1.513)	1.142 (0.876–1.490)	1.190 (0.922–1.535)
Q3 (10.7 μg/m^3^)	2.408^b^ (1.508–3.845)	2.302^b^ (1.474–3.597)	2.307^b^ (1.484–3.586)	2.295^b^ (1.476–3.568)	2.317^b^ (1.498–3.582)	2.234^b^ (1.467–3.404)	1.624^b^ (1.129–2.336)	1.366^a^ (1.007–1.853)	1.469^b^ (1.099–1.963)
Q4 (13.4 μg/m^3^)	2.797^b^ (1.439–5.436)	2.678^b^ (1.414–5.070)	2.697^b^ (1.433–5.074)	2.682^b^ (1.424–5.052)	2.680^b^ (1.433–5.012)	2.596^b^ (1.416–4.760)	1.712^a^ (1.013–2.893)	1.141 (0.734–1.776)	1.140 (0.749–1.736)
Annual average Temperature over 12 years	0.919 (0.835–1.012)	0.914 (0.835–1.001)	0.914^a^ (0.836–1.000)	0.915 (0.837–1.001)	0.918 (0.841–1.003)	0.921 (0.846–1.004)	0.903^b^ (0.839–0.972)	0.867^b^ (0.814–0.923)	0.855^b^ (0.805–0.908)
Number of intensive care and resuscitation beds (per 100,000)	1.015 (0.991–1.039)	1.010 (0.988–1.033)	1.009 (0.987–1.031)	1.008 (0.986–1.031)	1.008 (0.986–1.030)	1.006 (0.985–1.027)	1.006 (0.987–1.024)	1.000 (0.984–1.015)	1.001 (0.986–1.017)
Medical density (per 100,000)	0.998 (0.996–1.001)	0.999 (0.997–1.001)	0.999 (0.997–1.001)	0.999 (0.997–1.002)	0.999 (0.997–1.002)	1.000 (0.998–1.002)	1.000 (0.998–1.002)	1.001 (0.999–1.002)	1.001 (0.999–1.002)
% People aged 60 or more	1.073^a^ (1.009–1.140)	1.078^a^ (1.017–1.144)	1.080^b^ (1.019–1.145)	1.083^b^ (1.021–1.148)	1.079^a^ (1.018–1.143)	1.079^b^ (1.020–1.141)	1.032 (0.986–1.081)	1.017 (0.977–1.059)	1.013 (0.975–1.053)
% Males	1.436 (0.986–2.092)	1.494^a^ (1.040–2.147)	1.530^a^ (1.068–2.191)	1.561^a^ (1.091–2.235)	1.559^a^ (1.093–2.223)	1.543^a^ (1.092–2.180)	1.456^a^ (1.080–1.963)	1.308^a^ (1.010–1.694)	1.278 (0.997–1.637)
% Unemployment	1.027 (0.889–1.186)	1.014 (0.884–1.163)	1.015 (0.886–1.163)	1.015 (0.886–1.164)	1.020 (0.891–1.167)	0.989 (0.870–1.126)	0.997 (0.891–1.115)	1.008 (0.914–1.111)	1.014 (0.924–1.114)
Rate of Poverty (per cent)	1.000 (0.910–1.100)	1.000 (0.914–1.094)	1.002 (0.917–1.096)	1.002 (0.916–1.095)	1.000 (0.916–1.093)	1.018 (0.935–1.109)	1.017 (0.944–1.095)	1.006 (0.944–1.072)	1.003 (0.944–1.065)
% Urban population (proportion of people living in the great urban areas)	1.025^b^ (1.009–1.040)	1.024^b^ (1.009–1.039)	1.024^b^ (1.010–1.039)	1.025^b^ (1.010–1.040)	1.024^b^ (1.010–1.039)	1.025^b^ (1.011–1.039)	1.014^a^ (1.003–1.026)	1.009 (0.999–1.019)	1.006 (0.996–1.015)
Population density (inhab/square)	1.000^b^ (1.000–1.000)	1.000^b^ (1.000–1.000)	1.000^b^ (1.000–1.000)	1.000^b^ (1.000–1.000)	1.000^b^ (1.000–1.000)	1.000^a^ (1.000–1.000)	1.000^a^ (1.000–1.000)	1.000^a^ (1.000–1.000)	1.000^a^ (1.000–1.000)
Standardized Prevalence of Diabetes (%)	1.191 (0.831–1.708)	1.235 (0.879–1.735)	1.247 (0.890–1.745)	1.253 (0.895–1.753)	1.250 (0.897–1.741)	1.244 (0.901–1.717)	1.288 (0.973–1.705)	1.353^a^ (1.066–1.716)	1.352^b^ (1.077–1.698)
*Criteria for assessing goodness of fit:*									
*Deviance*	*1.1782*	*1.2091*	*1.2114*	*1.2134*	*1.2086*	*1.2147*	*1.1827*	*1.1982*	*1.1927*
*Scaled Deviance*	*1.1782*	*1.2091*	*1.2114*	*1.2134*	*1.2086*	*1.2147*	*1.1827*	*1.1982*	*1.1927*
*Pearson Chi-Square*	*1.2419*	*1.2275*	*1.2168*	*1.2148*	*1.2164*	*1.1875*	*1.1898*	*1.1747*	*1.1834*
*Scaled Pearson X2*	*1.2419*	*1.2275*	*1.2168*	*1.2148*	*1.2164*	*1.1875*	*1.1898*	*1.1747*	*1.1834*
*BIC (smaller is better)*	*733.6727*	*769.6559*	*775.9792*	*779.4617*	*781.455*	*789.3626*	*813.6141*	*885.5104*	*927.3749*

(^a^) Significance at 5%, (^b^) Significance at 1%

For the association between the COVID-19 mortality rates and temperature, the results were stable and significant from 1 November.

In addition, the results showed that the adjustment variables related to the availability of health resources and socio-economic factors did not have an overall influence on the COVID-19 mortality rate between departments. In contrast, demographic variables and urbanisation rates were statistically associated with the COVID-19 mortality rate for several months.

## Discussion

This study provides significant findings on the associations between COVID-19 mortality rates, long-term exposure to PM_2.5_, and temperature in the context of socio-spatial disparity across the ninety-six departments of France. Our analyses mainly focus on the associations between, on one hand, long-term exposure to PM_2.5_ and COVID-19 mortality rate, and on the other hand, temperature and COVID-19 mortality rate.

### Spatial distribution of COVID-19 mortality rate and environmental factors

The geographic distribution of annual average PM_2.5_ concentration levels across France was uneven and reflected the distribution of emission sources and urbanisation rate of each department.

Our results showed an increasing trend in the distribution of COVID-19 mortality rates across the departments grouped into quartiles of the annual average level of PM_2.5_. Indeed, despite the gradual spread of the COVID-19 pandemic across the whole country, the mortality rate followed a gradient determined by the level of PM_2.5_, as shown in Fig. 1. They were all higher in the departments where the annual average of long-term PM_2.5_ concentrations was also high. Our results highlighted the existence of a strong correlation between the mortality rate due to COVID-19 and long-term exposure to PM_2.5_.

These results overlapped well with the COVID-19 incidence data published regularly by the Ministry of Health. Considered separately, some departments could present an epidemiological picture different from the group to which it was assigned based on its level of PM_2.5_ concentration. The spatial distribution of COVID-19 cases and deaths is not random and may be related to environmental factors [32].

These geographic disparities in COVID-19 mortality rates, depending on the level of long-term PM_2.5_, could be related to other parameters such as the pandemic's spatial dynamics, the intensity of social interactions, and aggregation of infected individuals [33]. These disparities could also be associated with the differential vulnerability between each sub-national level, such as the existence of medical conditions [2, 27] and socio-economic conditions. Environmental factors and various vulnerabilities could represent a favourable context for the spread of the COVID-19 pandemic and the increase in COVID-19 deaths [17]. The maps made it possible to observe the evolution (or trend) of the COVID-19 mortality rate across departments, given their levels of long-term exposure to PM_2.5_, expressed in quartiles.

Other studies have shown significant correlations between long-term exposure to PM_2.5_, and the incidence or mortality due to the COVID-19 pandemic [17, 34]. Pansini and Fornacca [34] concluded that higher mortality was also correlated with poor air quality, namely, high PM_2.5_. In this new study, significant positive correlations of the COVID-19 mortality rate were found with long-term exposure to PM_2.5_, urbanisation rate, population density, and standardised prevalence rate of diabetes. In contrast, negative correlations were found with long-term annual average temperature.

### Association between COVID-19 mortality rate and long-term PM_2.5_ exposure

The existence of an association between the risk of death and hospitalisation for COVID-19 and long-term exposure to PM_2.5_ indicates the role of air pollution in the development of an increased vulnerability of specific populations to COVID -19, especially the elderly, men, and people living in large urban areas. We observed a positive gradient in the COVID-19 mortality rate linked to the annual average level of PM_2.5_, when defined by quartile.

Our study showed the stability of the impact of long-term exposure to PM_2.5_ on the COVID-19 mortality rate, varying from 1.244 (or 24.4%) to 1.258 (or 25.8%) on May 1 to October 1, respectively, before falling to 1.151 (or 15.1%) on 1 November then ceased to be statistically significant on December 1 and 31 2020. The beginning of the decrease in the intensity of the association between long-term exposure to PM_2.5_, and the COVID-19 mortality rate seemed to coincide with the pandemic's resurgence in September (see Supplementary Material Fig. 1), also called the second wave. This second wave amplified the spread of SARS-CoV-2 through departments that were slightly affected and had lower PM_2.5_. This would have gradually reduced the specific effects of geographic disparities in pollution levels between departments.

On the other hand, considering the distribution of long-term exposure to PM_2.5_, our results revealed a positive gradient in the impact of air pollution on the COVID-19 mortality rate from the third quartile. This gradient was observed until 1 November 2020. From December 1, only the departments belonging to the third quartile of the PM_2.5_ concentration level had higher COVID-19 mortality rates than those in the first quartile. This result is important as it suggests that the effect of long-term exposure to PM_2.5_ on the COVID-19 mortality rate was not significant in France only until the end of 2020. In addition, this effect remained associated with the geographic disparities in mortality rates observed when less comprehensive analyses could be performed. It should also be noted that there was no statistically significant difference in the COVID-19 mortality rates between the departments located in the first and the second quartiles. Since the start of the SARS-CoV-2 pandemic, several studies have advanced various hypotheses that may help to understand the mechanisms by which exposure to PM_2.5_, and air pollution in general, would influence the spread of the coronavirus and would induce the severity of infected cases and deaths [35–38]. For example, Wang et al. [39] considered that PM_2.5_ could facilitate SARS-CoV-2 infection through the overregulation of ACE2. As the impact of air pollution on health is well documented, the association between long-term air pollution and the severity of infected cases and mortality is thought to be mediated by the various morbidity conditions caused by chronic exposure to poor air quality.

This association could be explained by the fact that short- and long-term exposure to air pollution in the population was linked to asthma attacks, exacerbation of COPD, acute respiratory inflammation, and cardiorespiratory disease linked to death. Prolonged exposure to air pollution leads to a chronic inflammatory stimulus, even in young and healthy subjects, and could induce persistent modifications of the immune system, for which short-term changes in air quality may not be sufficient to break the aforementioned vicious circle [24]. Of course, the latter authors have observed the persistence of a high fatality rate, despite the dramatic reduction in air pollution levels in Lombardy since the start of the outbreak. Our results showed certain stability of the effects of long-term exposure to PM_2.5_, on the COVID-19 mortality rates. These results are consistent with those obtained by other studies in the USA, Italy, China, Great Britain, the Netherlands, and Spain.

In the USA, Wu et al. [13] found that an increase of only 1 μg/m^3^ in long-term average PM_2.5_, is associated with a statistically significant increase of 15% in the COVID-19 mortality rate with data collected on 5 April 2020. Using data from 18 June 2020 the same authors found that the association led to an increase of 11% in the COVID-19 mortality rate [31].

In the Netherlands, Cole et al. [40] examined COVID-19 data between February and June 2020 and found a statistically significant positive relationship between long-term PM_2.5_ exposure and COVID-19 deaths. Their findings indicated that an increase in PM_2.5_ concentrations of 1 μg/m^3^ was associated with an increase in COVID-19 deaths of between 13.0% and 21.4%.

Coker et al. [15] found a 1 μg/m^3^ increase in PM_2.5_, leading to a 9% increase in COVID-19 related excess mortality at the municipality level.

In a study concerning the regional and global contributions of air pollution to the risk of death from COVID-19, Pozzer et al. [37] found that the COVID-19 mortality fraction attributed to air pollution was 11% for fossil fuel-related emissions in France, 17% in Germany, 12% in Italy, and 9% in the UK. These authors considered that long-term exposure to high levels of PM_2.5_ is a significant co-factor that influences the severity of COVID-19 outcomes and increases the risk of mortality from SARS-CoV-2 [37].

However, the scope of these significant associations should be moderated because the health outcome considered, whether the number of cases of COVID-19 or the number of deaths, was measured during the first 2–3 months of the onset pandemic. It is a short period, during which the spread of the pandemic was mainly limited to parts of the countries, particularly because of the adoption of containment and physical distancing measures. We assume that the magnitude of the association between long-term exposure to PM_2.5_, and the COVID-19 mortality rate would tend to decrease or even disappear as the spread of the pandemic progresses and affects multiple geographic areas across the entire country. Our study has shown how this association is sensitive to the spread of the pandemic over different periods. Indeed, in the case of France, with COVID-19 mortality data as of 1 November 2020 a 1 μg/m^3^ increase in PM_2.5_ corresponded to a 15% increase in COVID-19 mortality rate, while with the data from the month of December, this association was no longer significant with the same variables. This result suggests that the association was not sustainable in the long term, and it might only represent a snapshot of the spread of the pandemic in the country [41].

### Association between COVID-19 mortality rate and temperature

Several recent studies have analysed the influence of air quality on the spread of the COVID-19 pandemic and the resulting deaths [14, 18–20, 42–44]. Most of the results of these studies suggest that temperature could play an important role in the spread of the COVID-19 pandemic, as in the case of the influenza epidemic. On the contrary, other studies have not found statistically significant associations between the incidence and/or mortality due to COVID-19 and temperature [43].

Our results highlight the existence of these two seemingly contradictory situations during the analysis period. In fact, until 1 October 2020 the COVID-19 mortality rate was not statistically associated with temperature. However, from 1 November, the association became significant.

Over the study period, the multiplication coefficient of the association between mortality rate and temperature was less than 1, suggesting an inverse relationship between the two. However, this inverse relationship was statistically significant only with regards to the readings on 1 November, 1 December, and 31 December.

Thus, a degree of increase in the average temperature was associated with a 9.6% decrease in the COVID-19 mortality rate on 1 November, 13.0% a month later on 1 December, and then 14.1% as of 31 December 2020. These results could be explained by the fact that the COVID-19 mortality rate increased more sharply in the departments where low temperatures were more frequent than in the departments of the West and South-West of France.

Qi et al. [42] showed that both temperature and humidity are negatively associated with COVID-19.

In France, the emergence of the second wave coincided with the gradual drop in temperatures, notwithstanding the supposed relaxation of certain social distancing gestures, and tends to reinforce the hypothesis of a significant effect of temperature on the incidence and death rate of COVID-19. Our results are consistent with those found in China [18] and Spain in the Barcelona region [19]. Tobias and Molina [19] found that a 1 °C increase in maximum temperature reduced the incidence rate by 7.5% on the same day. Holtman et al. [20] found that ambient temperature plays a significant role in the spread of COVID-19 by promoting the survival of the virus in the environment when temperatures are low. Wang et al. [14] reported that low temperature and low humidity significantly contributed to the transmission and survival of coronaviruses. For these authors, ‘strict public health strategies should be continued when temperature drops in most parts of the country so as to prevent reversal of the epidemic’. Coker et al. [15] found a negative association between temperature and COVID-19 mortality. In contrast, Sobral et al. [43] found no significant correlation between the COVID-19 mortality rates and temperature.

Finally, our study confirms and extends the results of previous studies concerning the impact of environmental factors on the COVID-19 mortality rate.

The long observation period of cumulative deaths due to the COVID-19, from March to December 2020, has indeed made it possible to highlight the influence of the duration and extent of the spread of the COVID-19 on most of the results of previous studies. Typically, the significant positive association between the COVID-19 mortality rate and long-term exposure to PM_2.5_ did not cease until 1 December 2020, while the relationship between the mean annual temperature and COVID-19 mortality rate did not appear until 1 November 2020. This last period coincided with what many specialists called the second wave of the pandemic, which was observed in several European countries (such as the UK, Germany, Spain, Belgium, Portugal, and Italy). This second wave gained momentum as the temperature gradually dropped, resulting in more deaths than the first wave. Future research with data from other countries is needed to evaluate the consistency of the relationships observed in France between COVID-19 mortality and environmental factors. Likewise, the results of our study encourage us to pursue investigations with individual data when they are available and publicly accessible.

### Limitations

This study presents some inherent limitations to the ecological analysis, and our findings should be interpreted with caution due to some potential biases related to ecological data. First, within departments, long-term exposure to PM_2.5_ and COVID-19 deaths could vary from place to place, masking the heterogeneity within them. Second, the lack of individual data on COVID-19 deaths did not allow investigation of the precise long-term impact. In addition, the cross-sectional design of this study and the constraint of available data could also constitute limitations, as it is difficult to predict the evolution of the associations observed with time and the duration of the coronavirus pandemic itself. Furthermore, the COVID-19 pandemic is ongoing; hence, the study findings could change. Hence, the study results these should be considered intermediate results.

## Conclusions

This study found significant associations between the COVID-19 mortality rate, long-term exposure to PM_2.5_, and temperature. Due to the health implications of well-documented air pollution, it could play a role as a co-factor in COVID-19 mortality through induced comorbidities. However, the influence of these associations tended to decrease with the evolution of the pandemic and massive spread of the disease across the country. Our results suggest that long-term exposure to PM_2.5_, even at concentrations lower than those set by the EU guidelines set (25 μg/m^3^), has a severe impact on the state of health of the population; it has a strong association with the COVID-19 mortality rate. Public authorities should implement effective strategies to improve air quality to at least approach the limits set by the WHO at 10 μg/m^3^. The analysis at the sub-national level makes it possible to identify the determinants of geographic inequalities in COVID-19 mortality rates and help policymakers, clinicians, and public health practitioners understand the spatial distribution of the pandemic and adapt intervention strategies for current or future pandemics.

## Supplementary Information

**Additional file 1: Supplementary Table 1**. Availability of healthcare services and socio-spatial characteristics at the department level. **Supplementary Table 2**. Classification of departments in France by quartile of annual mean PM_2.5_ concentration level, in period 1999-2016 (by µg/m^3^). **Supplementary Table 3**: Relationships between COVID-19 mortality rate and exposure to PM2.5 and temperature. Unadjusted negative binomial regression analyses. **Supplementary Figure 1**: Daily evolution of the COVID-19 deaths in hospital in metropolitan France from 19 March 2,020 to 31 December, 2020

## Data Availability

We used publicly available data and have referenced the sources in the paper.
