# Supplementary material for: Impact of long-term exposure to PM2.5 and temperature on coronavirus disease mortality: observed trends in France
Source: Environ Health. 2021 Sep 6;20:101. doi: 10.1186/s12940-021-00784-1 (PMC8420152; doi:10.1186/s12940-021-00784-1)
Supplement: Supplementary file 1 — Additional file 1: Supplementary Table 1. Availability of healthcare services and socio-spatial characteristics at the department level. Supplementary Table 2. Classification of departments in France by quartile of annual mean PM2.5 concentration level, in period 1999-2016 (by µg/m3). Supplementary Table 3: Relationships between COVID-19 mortality rate and exposure to PM2.5 and temperature. Unadjusted negative binomial regression analyses. Supplementary Figure 1: Daily evolution of the COVID-19 deaths in hospital in metropolitan France from 19 March 2,020 to 31 December, 2020 [file 12940_2021_784_MOESM1_ESM.docx]

**Supplementary Appendix**

Supplementary to

“**Impact of long-term exposure to PM2.5 and temperature on COVID-19 mortality: observed trends in France”**

Anatase Tchicaya, PhD^1^ *, Nathalie Lorentz, MSC ^1^, Hichem Omrani, PhD^1^, Gaetan de Lanchy, MSC^1^, Kristell Leduc, MSC^1^

1. Luxembourg Institute of Socio-Economic Research (LISER), Living Conditions Department, 11 Porte des Sciences, L-4366 Esch-sur-Alzette, Luxembourg

***** Correspondence: [anastase.tchicaya@liser.lu](mailto:anastase.tchicaya@liser.lu) Tel.: +352-58-58-55-521.

[Supplementary Tables 2](#_Toc79146726)

[Supplementary Table 1: Availability of healthcare services and socio-spatial characteristics at the department level 2](#_Toc79146727)

[Supplementary Table 2: Classification of departments in France by quartile of annual mean PM_2.5_ concentration level, in period 1999-2016 (by µg/m^3^) 8](#_Toc79146728)

[Supplementary Figures 10](#_Toc79146729)

[Supplementary Figure 1: Daily evolution of the COVID-19 deaths in hospital in metropolitan France from 19 March 2,020 to 31 December, 2020 10](#_Toc79146730)

# **Supplementary Tables**

## Supplementary Table 1: Availability of healthcare services and socio-spatial characteristics at the department level

| **Department** | | **Region name** | **Number of resuscitation beds (per 100,000 people)** | **Number of intensive care beds (per 100,000 people)** | **Medical density (per 100,000 people)** | **Population density (per 100,000 people)** | **Population size (by thousand)** | **% People aged 60 or more** | **% men** | **% Unemployment** | **% Urban population** | **Rate of poverty (per cent)** |  |
| --- | --- | --- | --- | --- | --- | --- | --- | --- | --- | --- | --- | --- | --- |
| **number** | **name** |  |  |  |  |  |  |  |  |  |  |  | **Diabetes** |
| 1 | Ain | Auvergne-Rhône-Alpes | 22 | 10 | 186 | 114·0 | 657·0 | 24·2 | 49·2 | 5·9 | 86·7 | 10·7 | 4.5 |
| 2 | Aisne | Hauts-de-France | 34 | 55 | 230 | 71·4 | 526·1 | 27·9 | 48·9 | 11·4 | 69·8 | 18·9 | 6.3 |
| 3 | Allier | Auvergne-Rhône-Alpes | 24 | 28 | 285 | 45·1 | 331·3 | 35·6 | 47·8 | 8·8 | 67·2 | 15·5 | 5.3 |
| 4 | Alpes-de-Haute-Provence | Provence-Alpes-Côte d'azur | 6 | 0 | 304 | 23·9 | 165·2 | 34·3 | 48·8 | 9·8 | 41·6 | 16·4 | 4.3 |
| 5 | Hautes-Alpes | Provence-Alpes-Côte d'azur | 8 | 0 | 441 | 25·5 | 141·8 | 32·4 | 48·8 | 7·6 | 45·7 | 14·3 | 3.8 |
| 6 | Alpes-Maritimes | Provence-Alpes-Côte d'azur | 94 | 91 | 461 | 251·1 | 1079·4 | 31·3 | 47·2 | 8·5 | 98·6 | 15·7 | 4.6 |
| 7 | Ardèche | Auvergne-Rhône-Alpes | 8 | 0 | 242 | 59·1 | 326·9 | 32·7 | 48·8 | 9 | 63 | 14·4 | 4.6 |
| 8 | Ardennes | Grand-Est | 12 | 21 | 239 | 50·8 | 265·5 | 29·7 | 48·6 | 9·9 | 61·6 | 19·4 | 6.0 |
| 9 | Ariège | Occitanie | 8 | 8 | 284 | 31·2 | 152·4 | 34·4 | 49·0 | 10·3 | 35·1 | 18·5 | 4.7 |
| 10 | Aube | Grand-Est | 12 | 18 | 234 | 51·6 | 309·9 | 28·3 | 48·4 | 10·2 | 65·8 | 16·2 | 5.5 |
| 11 | Aude | Occitanie | 16 | 22 | 297 | 60·7 | 372·7 | 34·1 | 48·0 | 10·9 | 60·9 | 21·1 | 5.0 |
| 12 | Aveyron | Occitanie | 11 | 16 | 267 | 31·9 | 278·4 | 35·3 | 49·3 | 6·2 | 34·4 | 13·7 | 3.9 |
| 13 | Bouches-du-Rhône | Provence-Alpes-Côte d'azur | 309 | 238 | 444 | 399·9 | 2034·5 | 26·9 | 47·8 | 9·6 | 98·8 | 18·5 | 5.5 |
| 14 | Calvados | Normandie | 99 | 64 | 367 | 124·6 | 691·5 | 29·1 | 47·9 | 7·5 | 88·7 | 12·6 | 4.4 |
| 15 | Cantal | Auvergne-Rhône-Alpes | 8 | 15 | 290 | 24·9 | 142·8 | 37·0 | 48·9 | 4·8 | 45 | 13·4 | 4.2 |
| 16 | Charente | Nouvelle-Acquitaine | 12 | 18 | 258 | 58·5 | 348·2 | 33·4 | 48·3 | 7·9 | 70·7 | 14·9 | 4.6 |
| 17 | Charente-Maritime | Nouvelle-Acquitaine | 30 | 36 | 311 | 94·3 | 647·1 | 35·9 | 47·8 | 8·3 | 68·8 | 13·4 | 4.3 |
| 18 | Cher | Centre-Val-de-Loire | 12 | 16 | 212 | 41·0 | 296·4 | 33·9 | 48·4 | 8·5 | 67·5 | 14·9 | 5.3 |
| 19 | Corrèze | Nouvelle-Acquitaine | 15 | 21 | 279 | 41·0 | 240·3 | 35·3 | 48·5 | 6·6 | 60·7 | 13·2 | 4.7 |
| 21 | Côte-d'Or | Bourgogne-Franche Comté | 63 | 95 | 323 | 60·8 | 532·9 | 27·9 | 48·1 | 6·5 | 84·7 | 11·6 | 4.8 |
| 22 | Côtes-d'Armor | Bretagne | 14 | 16 | 291 | 86·7 | 596·2 | 34·1 | 48·2 | 7·1 | 61 | 12·1 | 3.4 |
| 23 | Creuse | Nouvelle-Acquitaine | 8 | 8 | 389 | 20·9 | 116·3 | 39·3 | 48·6 | 7·7 | 27·5 | 18·4 | 5.2 |
| 24 | Dordogne | Nouvelle-Acquitaine | 19 | 18 | 266 | 45·1 | 408·4 | 37·8 | 48·3 | 8·4 | 47 | 16·5 | 4.6 |
| 25 | Doubs | Bourgogne-Franche Comté | 43 | 131 | 234 | 103·1 | 539·4 | 26·2 | 48·9 | 7·5 | 77·3 | 12·2 | 4.8 |
| 26 | Drôme | Auvergne-Rhône-Alpes | 24 | 34 | 238 | 79·7 | 520·6 | 28·9 | 48·4 | 9 | 73·4 | 15·2 | 4.8 |
| 27 | Eure | Normandie | 12 | 22 | 359 | 99·5 | 600·7 | 26·5 | 48·7 | 8·1 | 80·6 | 13 | 5.1 |
| 28 | Eure-et-Loir | Centre-Val-de-Loire | 20 | 22 | 298 | 73·0 | 429·4 | 27·7 | 48·8 | 7·8 | 78·4 | 12·2 | 5.3 |
| 29 | Finistère | Bretagne | 69 | 71 | 167 | 134·6 | 906·6 | 30·7 | 48·6 | 7 | 58·2 | 10·8 | 3.4 |
| 2A | Corse-du-Sud | Corse | 10 | 15 | 214 | 40·5 | 162·4 | 31·1 | 48·3 | 7·7 | 69·2 | 17·2 | 4.2 |
| 2B | Haute-Corse | Corse | 8 | 6 | 342 | 39·1 | 182·3 | 30·4 | 48·5 | 8·7 | 55·4 | 21·7 | 4.5 |
| 30 | Gard | Occitanie | 49 | 54 | 311 | 127·9 | 748·5 | 30·5 | 48·1 | 11·4 | 84·3 | 19·8 | 4.9 |
| 31 | Haute-Garonne | Occitanie | 165 | 205 | 424 | 222·1 | 1400·9 | 22·0 | 48·6 | 7·8 | 96·2 | 12·8 | 4.4 |
| 32 | Gers | Occitanie | 8 | 4 | 245 | 30·4 | 190·0 | 36·1 | 48·7 | 6·1 | 38·9 | 15 | 4.1 |
| 33 | Gironde | Nouvelle-Acquitaine | 195 | 131 | 422 | 163·7 | 1633·4 | 25·3 | 48·1 | 7·7 | 88·5 | 12·7 | 4.6 |
| 34 | Hérault | Occitanie | 128 | 171 | 440 | 192·8 | 1176·1 | 28·0 | 47·7 | 11·6 | 83·4 | 19·2 | 4.8 |
| 35 | Ille-et-Vilaine | Bretagne | 66 | 112 | 344 | 159·7 | 1082·1 | 23·9 | 48·8 | 6·2 | 91·4 | 10·6 | 3.2 |
| 36 | Indre | Centre-Val-de-Loire | 10 | 9 | 217 | 32·0 | 217·1 | 36·6 | 48·1 | 7·9 | 41·9 | 14·8 | 5.2 |
| 37 | Indre-et-Loire | Centre-Val-de-Loire | 86 | 58 | 382 | 98·8 | 605·4 | 28·6 | 48·0 | 7·5 | 84·4 | 12·8 | 4.6 |
| 38 | Isère | Auvergne-Rhône-Alpes | 82 | 89 | 344 | 170·2 | 1265·0 | 24·8 | 49·0 | 6·6 | 93·3 | 11·5 | 4.9 |
| 39 | Jura | Bourgogne-Franche Comté | 8 | 9 | 242 | 51·6 | 257·8 | 31·3 | 48·6 | 5·9 | 55·6 | 11·7 | 4.5 |
| 40 | Landes | Nouvelle-Acquitaine | 14 | 16 | 276 | 44·6 | 412·0 | 33·0 | 48·3 | 8 | 48·6 | 11·9 | 4.5 |
| 41 | Loir-et-Cher | Centre-Val-de-Loire | 12 | 8 | 242 | 51·7 | 327·8 | 32·3 | 48·5 | 6·7 | 61·9 | 12·8 | 5.1 |
| 42 | Loire | Auvergne-Rhône-Alpes | 78 | 60 | 339 | 160·0 | 764·7 | 28·8 | 48·3 | 8·1 | 88·5 | 14·9 | 5.0 |
| 43 | Haute-Loire | Auvergne-Rhône-Alpes | 8 | 10 | 226 | 45·6 | 226·9 | 32·2 | 49·2 | 6·5 | 51·1 | 12·3 | 4.4 |
| 44 | Loire-Atlantique | Pays-de-Loire | 87 | 131 | 344 | 211·1 | 1437·1 | 24·2 | 48·5 | 6·7 | 89·6 | 10·2 | 3.7 |
| 45 | Loiret | Centre-Val-de-Loire | 51 | 60 | 244 | 100·8 | 682·9 | 26·4 | 48·6 | 7·9 | 82·6 | 13·2 | 5.4 |
| 46 | Lot | Occitanie | 8 | 4 | 273 | 33·2 | 173·2 | 38·9 | 48·4 | 8·1 | 40·8 | 14·7 | 4.1 |
| 47 | Lot-et-Garonne | Nouvelle-Acquitaine | 11 | 18 | 252 | 61·6 | 330·3 | 33·9 | 48·0 | 8·3 | 64·3 | 17·2 | 4.5 |
| 48 | Lozère | Occitanie | 3 | 0 | 237 | 14·8 | 76·3 | 33·5 | 49·6 | 5·1 | 0·1 | 14·3 | 3.8 |
| 49 | Maine-et-Loire | Pays-de-Loire | 66 | 98 | 325 | 113·8 | 815·9 | 27·3 | 48·7 | 7·5 | 76·1 | 11·7 | 4.1 |
| 50 | Manche | Normandie | 28 | 26 | 255 | 82·6 | 490·7 | 33·0 | 48·7 | 6 | 50·3 | 12·5 | 4.0 |
| 51 | Marne | Grand-Est | 60 | 39 | 342 | 69·1 | 563·8 | 26·1 | 48·4 | 7·5 | 77·6 | 14·4 | 5.7 |
| 52 | Haute-Marne | Grand-Est | 8 | 6 | 259 | 27·3 | 169·3 | 33·1 | 48·9 | 6·9 | 49·7 | 15·6 | 5.5 |
| 53 | Mayenne | Pays-de-Loire | 8 | 8 | 200 | 59·0 | 305·4 | 29·5 | 49·2 | 5·4 | 54·9 | 11·7 | 3.5 |
| 54 | Meurthe-et-Moselle | Grand-Est | 95 | 86 | 403 | 139·2 | 730·4 | 26·2 | 48·7 | 7·8 | 92·3 | 14·5 | 5.5 |
| 55 | Meuse | Grand-Est | 8 | 10 | 213 | 29·2 | 181·6 | 30·8 | 49·5 | 7·5 | 53·2 | 15·5 | 5.3 |
| 56 | Morbihan | Bretagne | 22 | 32 | 305 | 110·7 | 755·6 | 32·5 | 48·4 | 7 | 67·2 | 11·3 | 3.5 |
| 57 | Moselle | Grand-Est | 88 | 56 | 273 | 166·6 | 1035·9 | 27·4 | 48·9 | 7·9 | 88·7 | 14·6 | 5.8 |
| 58 | Nièvre | Bourgogne-Franche Comté | 12 | 16 | 233 | 29·3 | 199·6 | 38·3 | 48·0 | 7 | 46 | 15·8 | 5.7 |
| 59 | Nord | Hauts-de-France | 239 | 349 | 358 | 450·8 | 2589·0 | 23·2 | 48·3 | 10·5 | 95·4 | 19·2 | 6.2 |
| 60 | Oise | Hauts-de-France | 36 | 46 | 217 | 140·8 | 825·1 | 23·7 | 48·8 | 8·1 | 91·5 | 13·1 | 5.8 |
| 61 | Orne | Normandie | 16 | 12 | 242 | 45·4 | 276·9 | 34·1 | 48·8 | 7·5 | 33·8 | 16 | 4.6 |
| 62 | Pas-de-Calais | Hauts-de-France | 85 | 137 | 255 | 217·8 | 1452·8 | 25·8 | 48·5 | 9·9 | 93·6 | 19·8 | 6.3 |
| 63 | Puy-de-Dôme | Auvergne-Rhône-Alpes | 81 | 52 | 363 | 82·8 | 660·2 | 28·8 | 48·4 | 7 | 81·6 | 12·9 | 4.7 |
| 64 | Pyrénées-Atlantiques | Nouvelle-Acquitaine | 37 | 55 | 386 | 89·4 | 683·2 | 31·2 | 48·0 | 6·8 | 77·3 | 12·1 | 4.0 |
| 65 | Hautes-Pyrénées | Occitanie | 12 | 22 | 352 | 50·8 | 226·8 | 35·4 | 48·2 | 9 | 52 | 15 | 4.3 |
| 66 | Pyrénées-Orientales | Occitanie | 32 | 32 | 342 | 116·4 | 479·0 | 33·7 | 47·4 | 13·3 | 83·7 | 20·7 | 4.7 |
| 67 | Bas-Rhin | Grand-Est | 134 | 104 | 405 | 238·2 | 1132·6 | 25·5 | 48·5 | 6·8 | 87·2 | 13·1 | 5.7 |
| 68 | Haut-Rhin | Grand-Est | 70 | 68 | 303 | 216·5 | 763·2 | 27·0 | 49·0 | 7·8 | 88 | 13·1 | 5.4 |
| 69 | Rhône | Auvergne-Rhône-Alpes | 222 | 211 | 448 | 577·4 | 1876·1 | 22·3 | 48·1 | 7·2 | 97·7 | 13·9 | 5.1 |
| 70 | Haute-Saône | Bourgogne-Franche Comté | 12 | 12 | 236 | 43·5 | 233·2 | 31·1 | 49·1 | 7·4 | 52·9 | 14 | 5.1 |
| 71 | Saône-et-Loire | Bourgogne-Franche Comté | 26 | 22 | 262 | 63·9 | 547·8 | 33·7 | 48·5 | 7·4 | 62·5 | 13 | 5.1 |
| 72 | Sarthe | Pays-de-Loire | 21 | 27 | 235 | 90·3 | 560·2 | 29·4 | 48·5 | 8·1 | 80·5 | 13·4 | 4.8 |
| 73 | Savoie | Auvergne-Rhône-Alpes | 18 | 18 | 367 | 71·8 | 432·5 | 27·9 | 48·8 | 5·9 | 73 | 10·1 | 3.9 |
| 74 | Haute-Savoie | Auvergne-Rhône-Alpes | 34 | 41 | 303 | 188·8 | 828·4 | 22·7 | 49·1 | 6 | 92 | 9·2 | 3.9 |
| 75 | Paris | Île-de-France | 471 | 541 | 858 | 20459·7 | 2148·3 | 22·6 | 47·0 | 6·2 | 100 | 15·8 | 4.4 |
| 76 | Seine-Maritime | Normandie | 103 | 150 | 328 | 198·1 | 1243·8 | 26·9 | 48·0 | 9·2 | 86·8 | 14·7 | 5.3 |
| 77 | Seine-et-Marne | Île-de-France | 77 | 50 | 214 | 240·7 | 1423·6 | 20·5 | 48·5 | 6·7 | 98·7 | 11·6 | 5.8 |
| 78 | Yvelines | Île-de-France | 84 | 102 | 287 | 634·2 | 1448·6 | 22·6 | 48·5 | 6·2 | 100 | 9·7 | 4.6 |
| 79 | Deux-Sèvres | Nouvelle-Acquitaine | 8 | 18 | 236 | 62·1 | 372·6 | 30·9 | 48·8 | 5·8 | 43·2 | 12·4 | 4.6 |
| 80 | Somme | Hauts-de-France | 66 | 82 | 356 | 92·3 | 569·8 | 26·7 | 48·5 | 9·5 | 64·9 | 17·1 | 6.0 |
| 81 | Tarn | Occitanie | 24 | 35 | 299 | 67·4 | 387·9 | 32·5 | 48·0 | 8·5 | 56·9 | 15·5 | 4.2 |
| 82 | Tarn-et-Garonne | Occitanie | 18 | 20 | 264 | 70·6 | 262·6 | 28·8 | 48·9 | 9·4 | 77·2 | 17·2 | 4.7 |
| 83 | Var | Provence-Alpes-Côte d'azur | 60 | 42 | 337 | 179·8 | 1073·8 | 33·3 | 47·9 | 8·9 | 87·9 | 15·5 | 4.6 |
| 84 | Vaucluse | Provence-Alpes-Côte d'azur | 16 | 30 | 333 | 157·3 | 561·0 | 29·0 | 48·0 | 10·6 | 79·9 | 19·7 | 5.3 |
| 85 | Vendée | Pays-de-Loire | 18 | 22 | 219 | 101·7 | 683·2 | 32·1 | 48·6 | 6·3 | 49·7 | 9·6 | 4.2 |
| 86 | Vienne | Nouvelle-Acquitaine | 56 | 62 | 353 | 62·6 | 437·4 | 29·2 | 48·1 | 6·5 | 79·4 | 14·1 | 4.4 |
| 87 | Haute-Vienne | Nouvelle-Acquitaine | 26 | 72 | 413 | 67·2 | 370·8 | 32·3 | 47·5 | 7·4 | 77·1 | 15·3 | 5.0 |
| 88 | Vosges | Grand-Est | 8 | 12 | 254 | 61·2 | 359·5 | 32·7 | 48·6 | 8·7 | 55·9 | 15·7 | 4.9 |
| 89 | Yonne | Bourgogne-Franche Comté | 18 | 16 | 222 | 44·7 | 332·1 | 32·2 | 48·8 | 7·6 | 59·5 | 14·7 | 5.5 |
| 90 | Territoire de Belfort | Bourgogne-Franche Comté | 25 | 20 | 327 | 230·1 | 140·1 | 27·2 | 49·5 | 8·8 | 89·7 | 14·6 | 5.0 |
| 91 | Essonne | Île-de-France | 97 | 82 | 250 | 731·4 | 1319·4 | 20·5 | 48·9 | 6·5 | 100 | 12·9 | 5.4 |
| 92 | Hauts-de-Seine | Île-de-France | 210 | 138 | 399 | 9169·1 | 1613·8 | 20·7 | 47·5 | 6·3 | 100 | 12·2 | 4.6 |
| 93 | Seine-Saint-Denis | Île-de-France | 100 | 109 | 258 | 7076·9 | 1670·1 | 16·7 | 49·3 | 10·4 | 100 | 28·6 | 7.9 |
| 94 | Val-de-Marne | Île-de-France | 176 | 169 | 385 | 5738·9 | 1406·0 | 20·5 | 48·1 | 7·2 | 100 | 16·7 | 5.5 |
| 95 | Val-d'Oise | Île-de-France | 60 | 65 | 254 | 1001·9 | 1248·4 | 19·3 | 48·3 | 8·2 | 100 | 16·8 | 6.5 |

Source: French Ministry of Health (06/2020)

## Supplementary Table 2: Classification of departments in France by quartile of annual mean PM_2.5_ concentration level, in period 1999-2016 (by µg/m^3^)

| **Q1** | **Q2** | **Q3** | **Q4** |
| --- | --- | --- | --- |
| ***Mean: 7.2*** | ***Mean: 9.0*** | ***Mean: 10.7*** | ***Mean: 13.4*** |
| ***Min: 6.1*** | ***Min: 8.0*** | ***Min: 9.7*** | ***Min: 11.7*** |
| ***Max: 8.0*** | ***Max: 9.7*** | ***Max: 11.6*** | ***Max: 16.3*** |
| Alpes-de-Haute-Provence | Allier | Ain | Aisne |
| Ariège | Alpes-Maritimes | Calvados | Ardennes |
| Aude | Ardèche | Cher | Aube |
| Aveyron | Bouches-du-Rhône | Côte-d'Or | Bas-Rhin |
| Cantal | Charente | Deux-Sèvres | Essonne |
| Corrèze | Charente-Maritime | Doubs | Eure |
| Corse du sud | Dordogne | Haute-Marne | Eure-et-Loir |
| Creuse | Drôme | Haute-Saône | Haut-Rhin |
| Côtes d'Armor | Gard | Indre | Hauts-de-Seine |
| Finistère | Gers | Indre-et-Loire | Loiret |
| Haute Corse | Gironde | Jura | Marne |
| Haute-Garonne | Haute-Savoie | Loir-et-Cher | Meuse |
| Haute-Loire | Haute-Vienne | Maine-et-Loire | Moselle |
| Hautes-Alpes | Ille-et-Vilaine | Mayenne | Nord |
| Hautes-Pyrénées | Isère | Meurthe-et-Moselle | Oise |
| Hérault | Loire | Nièvre | Paris |
| Landes | Loire-Atlantique | Orne | Pas-de-Calais |
| Lot | Lot-et-Garonne | Rhône | Seine-Maritime |
| Lozère | Manche | Sarthe | Seine-Saint-Denis |
| Morbihan | Savoie | Saône-et-Loire | Seine-et-Marne |
| Puy-de-Dôme | Tarn-et-Garonne | Territoire de Belfort | Somme |
| Pyrénées-Atlantiques | Var | Vienne | Val-d'Oise |
| Pyrénées-Orientales | Vaucluse | Vosges | Val-de-Marne |
| Tarn | Vendée | Yonne | Yvelines |

Supplementary Table 3: Relationships between COVID-19 mortality rate and exposure to PM2.5 and temperature. Unadjusted negative binomial regression analyses

| **Exposure variables** | **01 May** | **01 June** | **01 July** | **01 August** | **01 September** | **01 October** | **01 November** | **01 December** | **31 December** |
| --- | --- | --- | --- | --- | --- | --- | --- | --- | --- |
| Annual average Long-term PM_2.5_ exposure (µg/m^3^) | 1.333**  (1.257-1.414) | 1.321** (1.249-1.398) | 1.324** (1.252-1.401) | 1.325** (1.253-1.402) | 1.325** (1.253-1.401) | 1.309** (1.241-1.381) | 1.241** (1.186-1.298) | 1.159** (1.112-1.208) | 1.144** (1.096-1.194) |
| Annual average Long-term PM_2.5_ exposure (µg/m^3^) in quartile (ref. =Q1) |  |  |  |  |  |  |  |  |  |
| Q2 | 1.462  (0.987-2.166) | 1.466*  (1.008-2.132) | 1.451  (1.000-2.105) | 1.452  (0.999-2.110) | 1.446  (0.999-2.092) | 1.463*  (1.020-2.098) | 1.345  (0.979-1.847) | 1.335*  (1.002-1.777) | 1.282  (0.969-1.697) |
| Q3 | 3.694**  (2.526-5.401) | 3.519** (2.445-5.067) | 3.521** (2.451-5.058) | 3.530** (2.452-5.081) | 3.504** (2.445-5.022) | 3.275** (2.303-4.656) | 2.382** (1.743-3.255) | 1.884** (1.418-2.504) | 1.949** (1.476-2.575) |
| Q4 | 5.744**  (3.940-8.373) | 5.363** (3.734-7.701) | 5.390** (3.761-7.724) | 5.387** (3.751-7.736) | 5.356** (3.746-7.657) | 5.045** (3.557-7.156) | 3.621** (2.656-4.936) | 2.341** (1.763-3.109) | 2.114** (1.601-2.792) |
| Annual average Temperature over 12 years | 0.829**  (0.726-0.947) | 0.831** (0.732-0.943) | 0.828** (0.729-0.940) | 0.827** (0.728-0.939) | 0.830** (0.732-0.942) | 0.844** (0.748-0.953) | 0.865** (0.785-0.952) | 0.861** (0.802-0.924) | 0.843** (0.788-0.901) |

NB: Unadjusted univariate model. (*)Significance at 5%, (**) Significance at 1%

# **Supplementary Figures**

## Supplementary Figure 1: Daily evolution of the COVID-19 deaths in hospital in metropolitan France from 19 March 2,020 to 31 December, 2020

Source: French Ministry of Health (03/2020 – 12/2020)
